# Long Non-Coding LEF1-AS1 Sponge miR-5100 Regulates Apoptosis and Autophagy in Gastric Cancer Cells via the miR-5100/DEK/AMPK-mTOR Axis

**DOI:** 10.3390/ijms23094787

**Published:** 2022-04-26

**Authors:** Huimin Zhang, Jun Wang, Yundan Wang, Jiapeng Li, Lili Zhao, Tongcun Zhang, Xinghua Liao

**Affiliations:** Institute of Biology and Medicine, College of Life and Health Sciences, Wuhan University of Science and Technology, Wuhan 430000, China; huiminzhang@wust.edu.cn (H.Z.); 201818703005@wust.edu.cn (J.W.); wangyundan@126.com (Y.W.); jiapengli@wust.edu.cn (J.L.); LiLiZhao2022@126.com (L.Z.); zhangtongcun@wust.edu.cn (T.Z.)

**Keywords:** miR-5100, LEF1-AS1, DEK, autophagy, apoptosis

## Abstract

DEK and miR-5100 play critical roles in many steps of cancer initiation and progression and are directly or indirectly regulated by most promoters and repressors. LEF1-AS1 as a long non-coding RNA can regulate tumor development through sponge miRNA. The effect and regulatory mechanism of DEK on autophagy and apoptosis in gastric cancer (GC), and the role between miR-5100 and DEK or miR-5100 and LEF1-AS1 are still unclear. Our study found that DEK was highly expressed in gastric cancer tissues and cell lines, and knockdown of DEK inhibited the autophagy of cells, promoted apoptosis, and suppressed the malignant phenotype of gastric cancer. DEK regulates autophagy and apoptosis through the AMPK/mTOR signaling pathway. In addition, miR-5100 inhibits autophagy and promotes apoptosis in GC cells while LEF1-AS1 had the opposite effect. Studies have shown that miR-5100 acts by targeting the 3′UTR of DEK, and LEF1-AS1 regulates the expression of miR-5100 by sponging with mIR-5100. In conclusion, our results found that LEF1-AS1 and miR-5100 sponge function, and the miR-5100/DEK/AMPK/mTOR axis regulates autophagy and apoptosis in gastric cancer cells.

## 1. Introduction

The prevalence of gastric cancer (GC) has declined significantly over the past few decades. However, it remains an important disease and is the third leading cause of cancer death worldwide [1,2]. Most stomach tumors are stomach adenocarcinoma (STAD) (90–95%) [3]. The current treatment of choice for patients with GC are surgical resection and appropriate lymphadenectomy. Current evidence supports perioperative therapies to improve patient survival. Unfortunately, patients with unresectable, locally advanced, or metastatic disease are limited to life-prolonging palliative care options [4]. Therefore, it is urgent to explore the molecular mechanism of GC occurrence and development and find effective therapeutic targets.

DEK is a DNA-binding protein and a regulator of chromatin structure, which is involved in the regulation of many cellular processes. The processes of proliferation [5,6,7], differentiation [8], apoptosis [5,9], senescence [10,11], DNA repair [12,13,14], and maintenance of the stem cell phenotype [8] are included. DEK plays a crucial role in many steps of cancer initiation and progression and is directly or indirectly regulated by most promoting and repressing factors. DEK may act as a structural regulatory protein that regulates the expression and function of various human genes in tumor cells [15]. DEK is highly expressed in GC and is associated with poor prognosis [6,16]. DEK has been reported to promote the proliferation [17], migration and invasion [18,19] of GC cells, but the exact molecular mechanism of DEK as an inducer to promote the occurrence and development of GC has not been clearly explored. Whether DEK is involved in the apoptosis and autophagy of GC cells and the molecular mechanism involved have not been reported.

MicroRNAs (miRNAs) are a small family of noncoding RNAs that regulate the expression of target genes by binding to the 3-untranslation region (3-UTR) of mRNA, about one-third of human genes may be regulated by miRNAs, suggesting that miRNAs may play an important role in cellular regulation [20,21,22]. Many studies have shown that miRNAs regulate a series of basic biological, such as cell proliferation, differentiation and apoptosis. miRNAs play an important role in the occurrence and development of cancer [23,24,25,26]. mR-5100, a novel miRNA, holds promise as a potential new biomarker for the diagnosis of oral squamous cell carcinoma and as a promising prognostic predictor for oral squamous cell carcinoma [22,27]. Meanwhile, it has been reported that the proliferation and migration of lung cancer cells are associated with miR-5100 [23,28], and in pancreatic cancer, overexpression of miR-5100 can reduce the aggressive phenotype of cells by targeting PODXL [29]. The exact molecular mechanism of miR-5100 involved in GC cell apoptosis and autophagy still needs to be explored.

Long non-coding RNAs are a group of RNA molecules that lack protein-coding functions [30,31]. They are involved in a series of processes regulating tumor biology and play important roles in the regulation of oncogenes or tumor suppressor genes [32]. Abnormal expression of lncRNAs in a variety of cancers has been reported, suggesting that they play an important role in regulating cancer cell proliferation, chemoresistance, and metastasis [33]. LncRNAs are often used as competing endogenous RNAs (ceRNAs) to regulate gene expression by specifically sponge the corresponding microRNAs to establish a large regulatory system across the transcriptome [34,35]. The regulated target genes are widely involved in various signaling pathways, most of which are closely related to tumors [36]. LEF1 (lymphoid enhancer factor binding factor 1), a nuclear transcription factor, is normally expressed in T cells and pre-B cells [37]. LEF1 antisense RNA 1 (LEF1-AS1), a recently discovered lncRNA, is located on chromosome 4q25 and encodes the lymphoid enhancer binding factor 1 (LEF1) locus. LEF1 is highly expressed in ovarian cancer [38], lung cancer [39], liver cancer [40] and other cancers [41], and regulates the occurrence and development of tumors [42]. Our study found that LEF1-AS1 was highly expressed in GC tumor tissues and cell lines. Interestingly, we predicted in the database that LEF1-AS1 may act as a sponge with miR-5100, suggesting that LEF1-AS1 may affect GC development by regulating the expression level of miR-5100 in GC cells. In the manuscript, we focused on the effect of LEF1-AS1 on apoptosis and autophagy of GC cells and the exploration of the molecular mechanism.

The expression level of DEK in GC and its effect on GC cell autophagy and apoptosis were our main concern, and the molecular mechanism of DEK’s effect on GC cell autophagy and apoptosis was also discussed. We found that miR-5100, which may act as an upstream regulator of DEK, is also negatively regulated by LEF1-AS1. This study will conduct an in-depth discussion of this series of regulatory mechanisms, and provide theoretical support for the prediction of effective therapeutic targets for GC in the future.

## 2. Results

### 2.1. DEK Is Highly Expressed in GC Tissues and Cells

**Figure 1:**
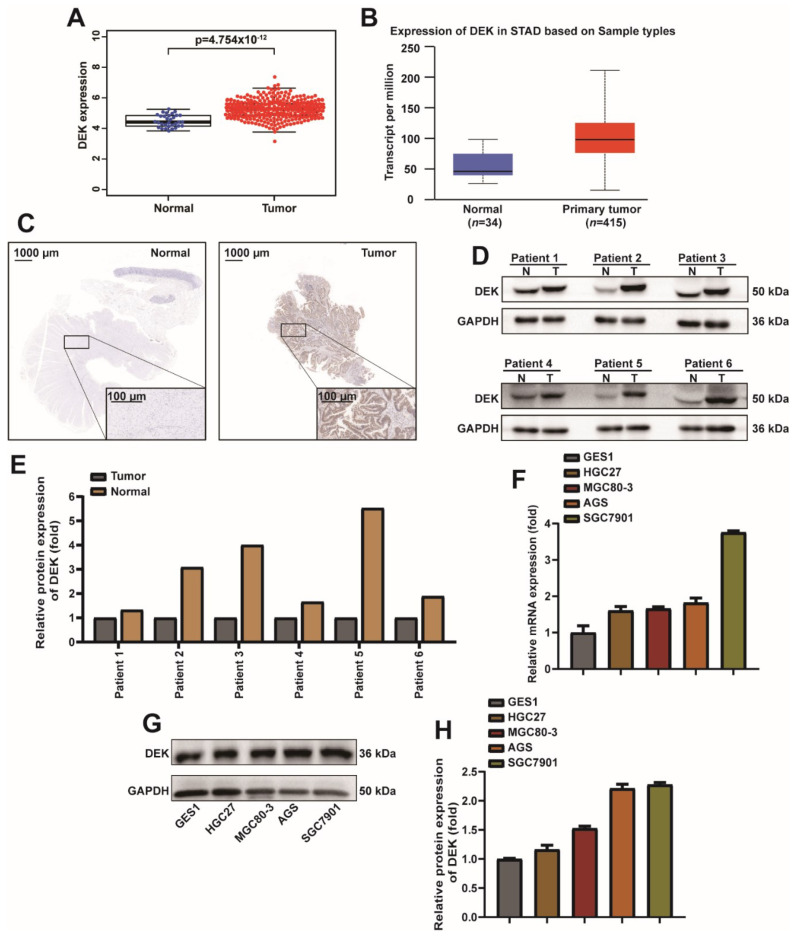
DEK is highly expressed in GC tissues and cells. (**A**) TCGA database analysis of DEK levels in GC tissues and normal tissues. (**B**) DEK expression levels in STAD are predicted at the UALCAN. (**C**) Representative images of DEK immunohistochemistry (IHC) in normal tissue of GC (left) and GC tissue (right). (**D**) Western Blot assay detected DEK protein in normal tissues and GC tissues in 6 pairs of samples. (**E**) The Western Blot signal for each patient was normalized with GAPDH and presented as a histogram. (**F**) qPCR experiments to analyze DEK mRNA levels in GES1, HGC27, MGC80-3, AGS, and SGC7901 cell lines and normalized to GAPDH. (**G**,**H**) Western blot assay to detect DEK protein expression in GES1, HGC27, MGC80-3, AGS, and SGC7901 cell lines. Western Blot signals were normalized with Actin. Among them, the differentiation degree of HGC27, MGC8-3, AGS and SGC7901 cells increased in turn.

In order to explore the expression pattern of DEK in GC, we analyzed the expression of DEK in GC (Figure 1A) in the TCGA database, and the same conclusion was found on the website (http://ualcan.path.uab.edu/cgi-bin/ualcan-res.pl, accessed on 19 October 2021) (Figure 1B). In order to further confirm the high expression of DEK in GC, 15 pairs of GC tissues and normal tissues were analyzed by IHC and Western Blot. IHC results showed that DEK was highly expressed in cancer tissues of patients compared with normal tissues (Figure 1C). Western Blot results and quantitative analysis showed that DEK was highly expressed in cancer tissues (Figure 1D,E). Next, we analyzed the mRNA and protein expression levels of DEK in normal human gastric mucosal epithelial cells (GES1) and GC cell lines (HGC27, MGC80-3, AGS, SGC7901). The experimental results showed that DEK was highly expressed at the mRNA and protein levels in GC cells (Figure 1F,G). The quantitative results of Western Blot also showed that DEK was highly expressed at the protein level in GC cell lines (Figure 1H). In conclusion, we observed a high expression of DEK in GC from bioinformatics analysis, patient, and cell lines.

### 2.2. DEK Accelerates the Phenotype of GC

**Figure 2:**
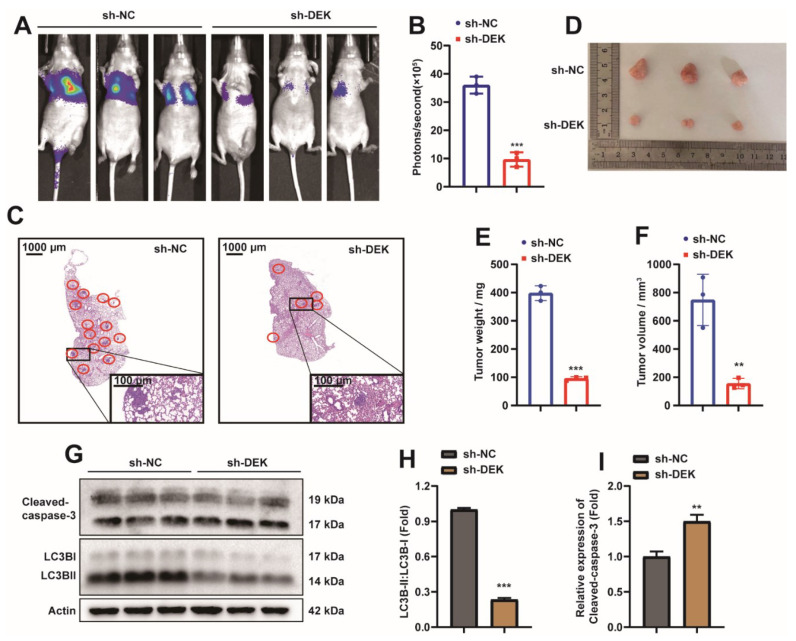
DEK accelerates the phenotype of GC. (**A**) Lung metastases were observed after luciferase-labeled DEK-knockdown (sh-DEK) or no-knockdown (sh-NC) SGC7901 cells were injected into nude mice via tail vein. (**B**) The luciferase bioluminescence values of mouse lung metastases (*n* = 3). (**C**) HE staining analysis of lung metastasis in nude mice. (**D**) Xenografts were obtained by subcutaneous injection of DEK-knockdown (sh-DEK) or no-knockdown (sh-NC) SGC7901 cells. (**E**,**F**) The weight and volume of subcutaneous xenografts were counted and presented in the form of histograms (**: *p* < 0.01, ***: *p* < 0.001). (**G**,**H**) Western Blot analysis in subcutaneous xenograft tissue LC3B expression, results were normalized with Actin, and LC3BII/I ratios were analyzed. (**H**,**I**) Western Blot analysis of Cleaved-caspase-3 protein in subcutaneous xenograft tissue and normalized with Actin (**: *p* < 0.01, ***: *p* < 0.001).

Given that DEK is highly expressed in GC cells, the role of DEK in GC was explored. Determination of the in vivo function of DEK in the BALB/c mouse animal model. First, we injected DEK-knockdown (sh-DEK) or no-knockdown (sh-NC) luciferase-labeled SGC7901 cells into nude mice via the tail vein, and then detected tumor growth by measuring the amount of bioluminescence (BLI). The knockdown efficiency of DEK was complemented in S1A. We found that knockdown of DEK significantly inhibited the metastatic ability of SGC7901 cells (Figure 2A,B). At the same time, we harvested the lung tissue of nude mice after 28 days, and found that knockdown of DEK could significantly reduce the metastatic foci in the lungs of nude mice by HE staining (Figure 2C). These findings were complemented by tumor xenografts in nude mice, where tumor size was measured after subcutaneous injection of the SGC7901 cell line with or without DEK knockdown (Figure 2D). Subcutaneous tumor growth was reduced in nude mice injected with knockdown DEK cells (Figure 2E,F). Taken together, these findings provide evidence that DEK accelerates GC metastasis and growth. Western Blot results showed that the ratio of LC3BII/I decreased in the xenografts of DEK knockout (Figure 2G,H), indicating that the level of autophagy in the knockdown DEK group was reduced, and the detection of cleaved-caspase3 expression indicated that the apoptosis of the DEK knockdown group increased (Figure 2G,I).

### 2.3. DEK Promotes Autophagy and Inhibits Apoptosis in GC Cells

**Figure 3:**
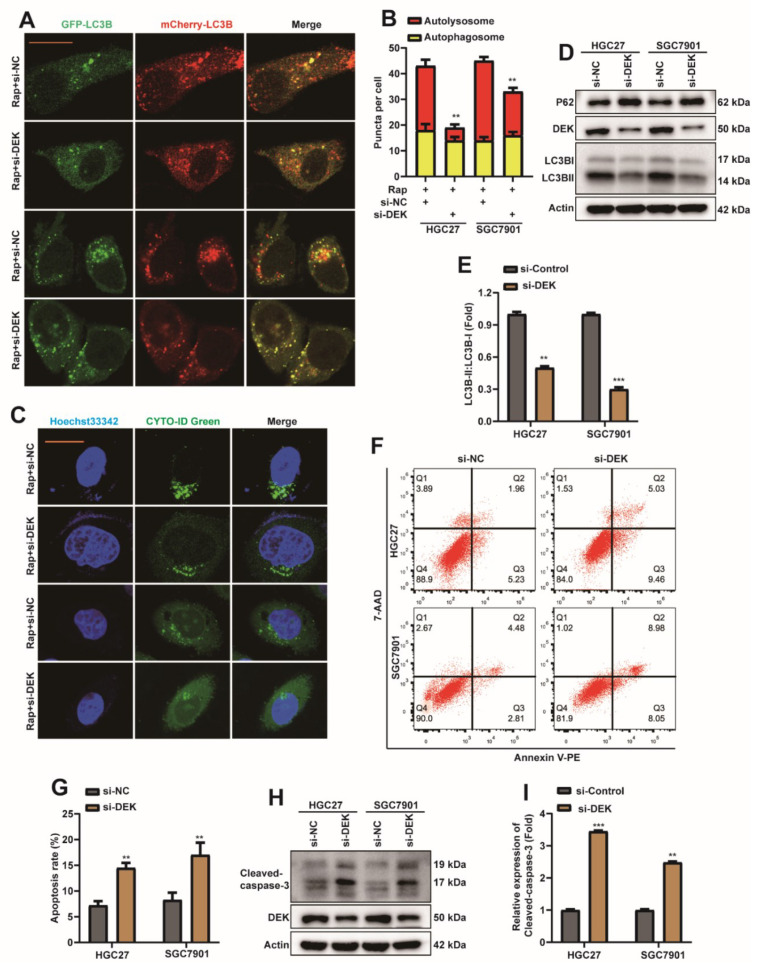
DEK promotes autophagy and inhibits apoptosis in GC cells. (**A**) si-DEK or si-NC were transfected into HGC27 or SGC7901 cell lines stably expressing mCherry-EGFP-LC3B. Cells were treated with 5 nM rapamycin (Rap) 24 h after transfection, and autophagic flux was observed 8 h later (scar bar = 40 μm) (**: *p* < 0.01). (**B**) Autophagosome (red dots) and Autolysosome (yellow dots) were counted and analyzed. (**C**) si-DEK or si-NC were transfected into HGC27 or SGC7901 cell lines. Cells were treated with 5 nM rapamycin (Rap) 24 h after transfection, and the level of autophagy was observed 8 h later. (**D**,**E**) si-DEK or si-NC were transfected into HGC27 or SGC7901 cell lines, and cells were harvested 36 h later. Western Blot detection of DEK, p62, LC3B proteins. The ratio of LC3BII/I was analyzed by normalization with Actin (*n* = 3, **: *p* < 0.01, ***: *p* < 0.001). (**F**,**G**) si-DEK or si-NC were transfected into HGC27 or SGC7901 cell lines, and the apoptosis rate was analyzed by flow cytometry after 36 h, and statistical analysis was performed. (**H**,**I**) si-DEK or si-NC was transfected into HGC27 or SGC7901 cell lines, and 36 h later, the expression of Cleaved-caspase-3 was detected by Western Blot, and quantitative analysis was performed (*n* = 3, **: *p* < 0.01, ***: *p* < 0.001).

To explore whether DEK is involved in autophagy and apoptosis in GC cells, DEK was silenced by siRNA in HGC27 and SGC7901 cells (Figure S1B,C). First, si-DEK or si-NC were transfected into HGC27 or SGC7901 cell lines, which stably express mCherry-EGFP-LC3B. Cells were then treated with 5 nM rapamycin (Rap), and autophagic flux was observed 8 h later. Rapamycin is a common inducer of autophagy [43]. Interestingly, autolysosomes were reduced in HGC27 and SGC7901 cells after DEK knockdown, suggesting that autophagic flux in cells is inhibited (Figure 3A,B). Previous reports showed that Cyto-ID stains autophagic cells through labeling autophagic compartments [44]. Next, the CYTO-ID^®^ Autophagy detection kit detects the level of autophagy, and the expression levels of autophagy-related proteins LC3B and autophagy receptor protein SQSTM1/p62 (p62) were detected by Western Blot. The data indicated that the level of autophagy decreased in cells after DEK knockdown (Figure 3C), while the protein level of p62 was increased (Figure 3D), and the ratio of LC3BII/I was decreased (Figure 3D,E). The above data suggest that silencing DEK can inhibit autophagy in GC cells.

AnnexinV-PE/7-AAD apoptosis detection kit and Western Blot were used to explore the apoptosis changes of HGC27 or SGC7901 cell lines after DEK was silenced. The results of flow cytometry showed that the apoptosis level of cells was increased after silencing DEK (Figure 3F,G), and the protein level of cleaved-caspase3 was increased at the same time (Figure 3H,I). Therefore, we preliminarily concluded that silencing DEK in GC cells can promote the level of apoptosis.

### 2.4. DEK Promotes Autophagy and Inhibits Apoptosis in GC through AMPK/mTOR Signaling Pathway

**Figure 4:**
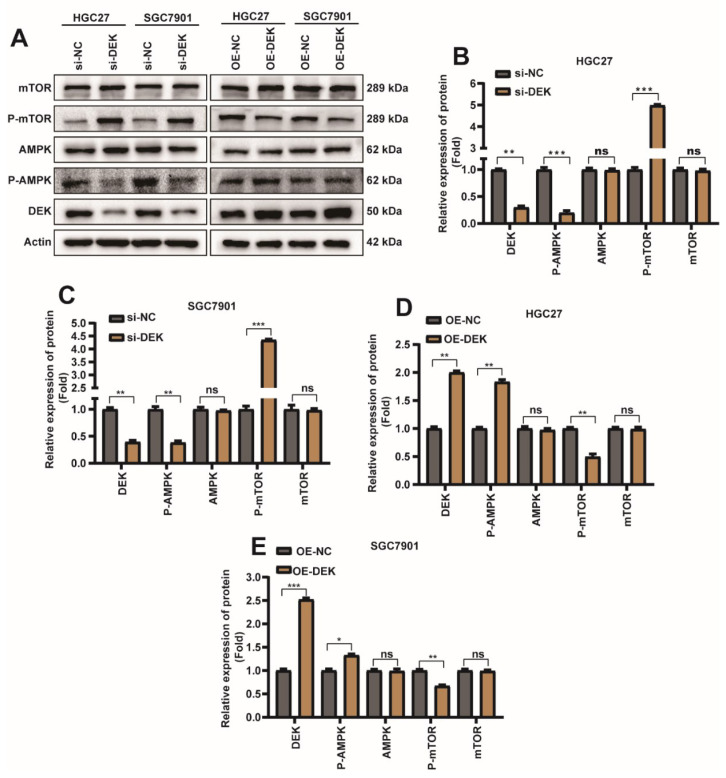
DEK promotes autophagy and inhibits apoptosis in GC through AMPK/mTOR signaling pathway. si-DEK/si-NC was transfected into HGC27/SGC7901 cell line, similarly OE-DEK/OE-NC was transfected into HGC27/SGC7901 cell line. The cells were harvested 36 h after transfection. (**A**) The protein expression levels of DEK, AMPK, p-AMPK, mTOR and p-mTOR were detected by Weather Blot. (**B**) Quantitative analysis of Weather Blot results for DEK, AMPK, p-AMPK, mTOR, and p-mTOR proteins after si-NC/si-DEK in HGC27 cells, normalized with Actin protein signal. (**C**) Quantitative analysis of Weather Blot results for DEK, AMPK, p-AMPK, mTOR, and p-mTOR proteins after si-NC/si-DEK in SGC7901 cells, normalized with Actin protein signal. (**D**) Quantitative analysis of Weather Blot results for DEK, AMPK, p-AMPK, mTOR and p-mTOR proteins after HGC27 cells were subjected to OE-NC/OE-DEK, normalized with Actin protein signal. (**E**) Quantitative analysis of Weather Blot results for DEK, AMPK, p-AMPK, mTOR and p-mTOR proteins after OE-NC/OE-DEK in SGC7901 cells, normalized with Actin protein signal (*n* = 3, *: *p* < 0.05, **: *p* < 0.01, ***: *p* < 0.001).

To explore the molecular mechanism of DEK regulating autophagy and apoptosis in GC cells, si-DEK/si-NC/DEK overexpression plasmid (OE-DEK)/control plasmid (OE-NC) was transfected into HGC27/SGC7901 cells. Next, we investigated the canonical signaling pathways that regulate autophagy and apoptosis. Surprisingly we found that in HGC27 or SGC7901 cells, overexpression or knockdown of DEK did not significantly affect the protein content of AMPK and mTOR, whereas knockdown of DEK decreased phosphorylated AMPK protein and increased phosphorylated mTOR protein (Figure 4A–C). When DEK was overexpressed, phosphorylated AMPK protein decreased and phosphorylated mTOR protein increased (Figure 4A,D,E). In conclusion, our study found that DEK can regulate autophagy and apoptosis in GC cells through the AMPK/mTOR signaling pathway.

### 2.5. miR-5100 Inhibits Autophagy and Promotes Apoptosis in GC Cells

**Figure 5:**
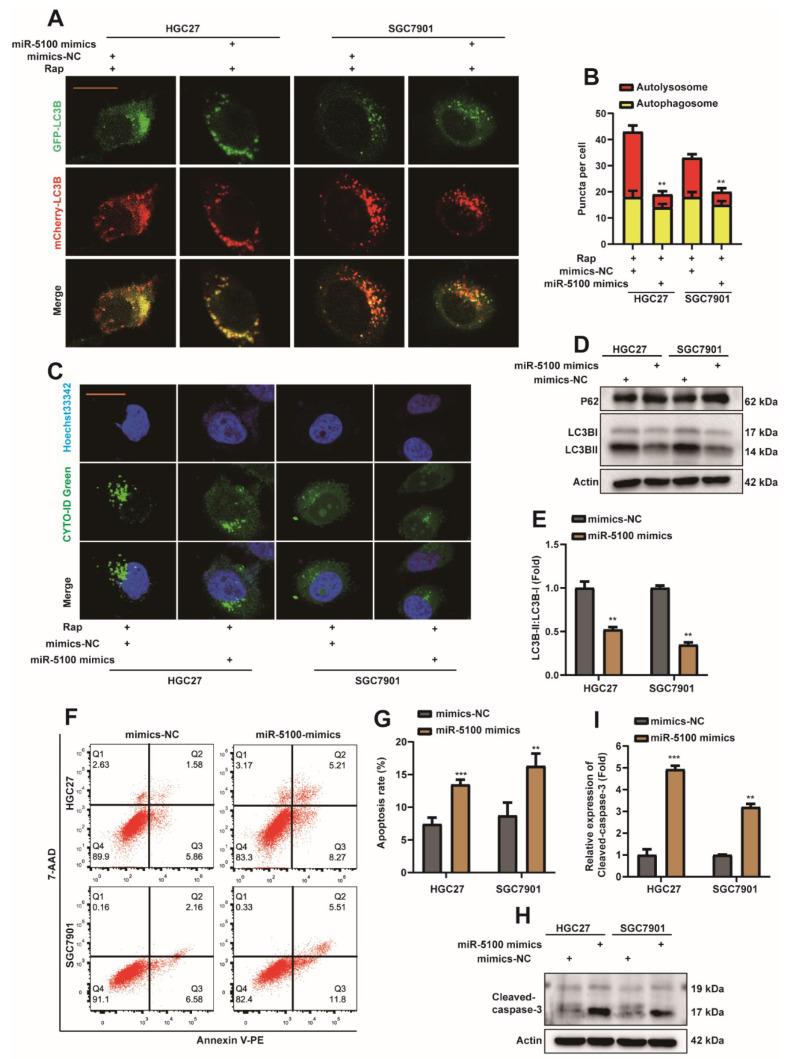
miR-5100 inhibits autophagy and promotes apoptosis in GC cells. (**A**) miR-5100 mimics/mimics-NC were transfected into HGC27 or SGC7901 cell lines stably expressing mCherry-EGFP-LC3B. Cells were treated with 5 nM rapamycin (Rap) 24 h after transfection, and autophagic flux was observed 8 h later (scar bar = 40 μm). (**B**) Autophagosome (red dots) and Autolysosome (yellow dots) were counted and analyzed, **: *p* < 0.01. (**C**) miR-5100 mimics/mimics-NCs were transfected into HGC27 or SGC7901 cell lines. Cells were treated with 5 nM rapamycin (Rap) 24 h after transfection, and the level of autophagy was observed 8 h later. (**D**,**E**) miR-5100 mimics/mimics-NCs were transfected into HGC27 or SGC7901 cell lines, and cells were harvested 36 h later. Western Blot detection of DEK, p62, LC3B proteins. The ratio of LC3BII/I was analyzed by normalization with Actin (*n* = 3, **: *p* < 0.01). (**F**,**G**) miR-5100 mimics/mimics-NC were transfected into HGC27 or SGC7901 cell lines, and the apoptosis rate was analyzed by flow cytometry after 36 h, and statistical analysis was performed (*n* = 3, **: *p* < 0.01, ***: *p* <0.001). (**H**,**I**) miR-5100 mimics/mimics-NC was transfected into HGC27 or SGC7901 cell lines, and 36 h later, the expression of Cleaved-caspase-3 was detected by Western Blot, and quantitative analysis was performed (*n* = 3, **: *p* < 0.01, ***: *p* <0.001).

miRNAs have been shown to be widely deregulated in human cancers, highlighting their important roles in tumor initiation, growth, and metastasis [45]. When we explored the mechanism of DEK overexpression in gastric cancer, we focused on the previously studied miR-5100. And to explore whether miR-5100 can regulate the autophagy and apoptosis of gastric cancer cells by affecting the expression of DEK. We found that the therapeutic effect of exosomes containing miR-5100 on gastric subcutaneous tumors was significantly better than that of miR-5100 liposomes (Figure S2). Briefly, we overexpressed miR-5100 in mesenchymal stem cells (MSCs) and extracted exosomes, and then observed the extracted exosomes under electron microscopy (Figure S2A). CD63, CD9, and CD81 are commonly used marker proteins of exosomes [46]. Next, the extracted exosomes were detected by Western Blot for exosome markers (Figure S2B), and the expression of miR-5100 in exosomes was detected by qPCR (Figure S2C). Animal experiments found that the therapeutic effect of exosomes on subcutaneous tumors was better than that of miR-5100-containing liposomes (Figure S2D), which was reflected in the mass and volume of subcutaneous tumors (Figure S2E,F). This phenomenon will be discussed in detail in the future. In this paper, we mainly focus on the effect of miR-5100 on the autophagy and apoptosis of GC cells. First, MiR-5100 mimics or mimics-NC were transfected into HGC27 or SGC7901 cell lines, which stably express mCherry-EGFP-LC3B. Interestingly, the autolysosome was reduced in HGC27 and SGC7901 cells in the transfected miR-5100 mimic group (Figure 5A,B). In HGC27 or SGC7901 cell lines transfected with miR-5100 mimic, the level of autophagy decreased (Figure 5C), and Western Blot detection showed that the protein level of p62 was increased (Figure 5D), and the ratio of LC3BII/I was decreased (Figure 5D,E). The above data indicate that miR-5100 can inhibit autophagy in GC cells. The results of flow cytometry showed that the level of cell apoptosis increased after transfection of miR-5100 mimic (Figure 5F,G), and the results of Western Blot showed that the level of cleaved-caspase3 protein increased (Figure 5H,I). Therefore, we tentatively concluded that miR-5100 could promote the level of apoptosis in GC cells.

### 2.6. Long Non-Coding LEF1-AS1 Promotes Autophagy and Inhibits Apoptosis in GC cells

**Figure 6:**
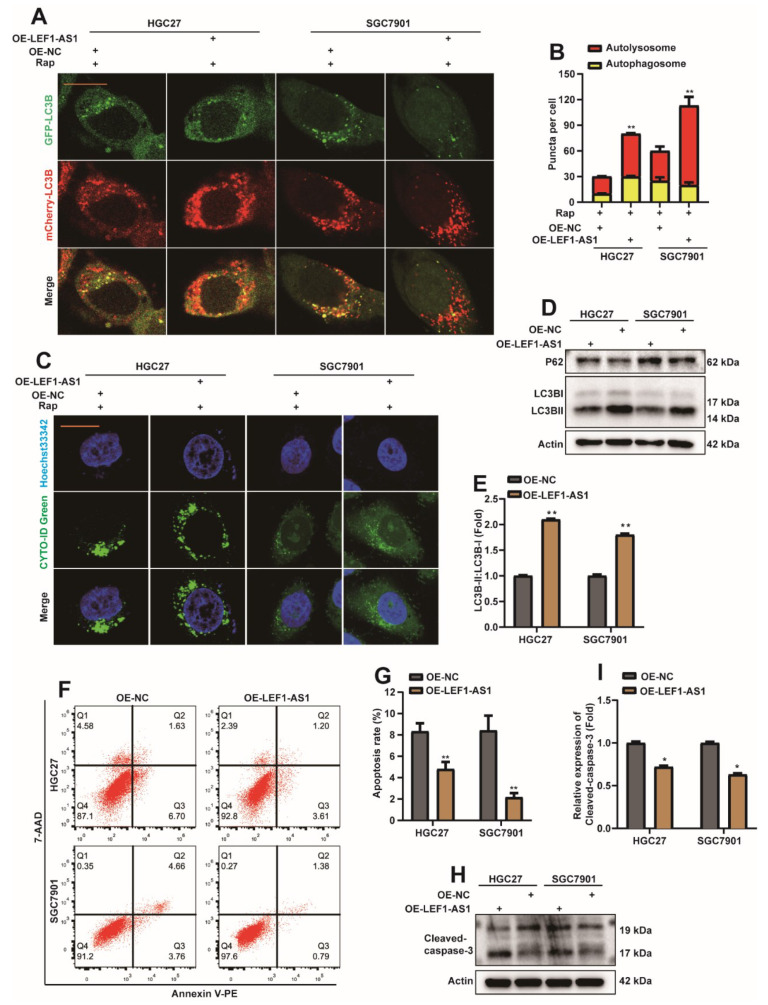
Long non-coding LEF1-AS1 promotes autophagy and inhibits apoptosis in GC cells. (**A**) OE-LEF1-AS1/OE-NC were transfected into HGC27 or SGC7901 cell lines stably expressing mCherry-EGFP-LC3B. Cells were treated with 5 nM rapamycin (Rap) 24 h after transfection, and autophagic flux was observed 8 h later (scar bar = 40 μm). (**B**) Autophagosome (red dots) and Autolysosome (yellow dots) were counted and analyzed (**: *p* < 0.01). (**C**) OE-LEF1-AS1/OE-NC were transfected into HGC27 or SGC7901 cell lines. Cells were treated with 5 nM rapamycin (Rap) 24 h after transfection, and the level of autophagy was observed 8 h later. (**D**,**E**) OE-LEF1-AS1/OE-NC were transfected into HGC27 or SGC7901 cell lines, and cells were harvested 36 h later. Western Blot detection of DEK, p62, LC3B proteins. The ratio of LC3BII/I was analyzed by normalization with Actin (*n* = 3, **: *p* < 0.01). (**F**,**G**) OE-LEF1-AS1/OE-NC was transfected into HGC27 or SGC7901 cell lines, and the apoptosis rate was analyzed by flow cytometry after 36 h, and statistical analysis was performed (*n* = 3, **: *p* < 0.01). (**H**,**I**) OE-LEF1-AS1/OE-NC was transfected into HGC27 or SGC7901 cell lines, and the expression of Cleaved-caspase-3 was detected by Western Blot 36 h later, and quantitative analysis was performed (*n* = 3, *: *p* < 0.05).

In addition, we also explored the effect of LEF1-AS1 on autophagy and apoptosis in GC cells. Similarly, overexpressing LEF1-AS1 plasmids or control plasmids were transfected into HGC27 or SGC7901 cell lines, which stably expressed mCherry-EGFP-LC3B. The results showed that overexpression of LEF1-AS1 increased the autolysosome in HGC27 and SGC7901 cells (Figure 6A,B). In addition, the experimental results of the CYTO-ID^®^ Autophagy detection kit showed that the level of autophagy increased significantly after the HGC27 or SGC7901 cell lines overexpressed LEF1-AS1 (Figure 6C). At the same time, Western Blot detection showed that the protein level of p62 was decreased (Figure 6D), and the ratio of LC3BII/I was increased (Figure 6D,E). After the overexpression of LEF1-AS1 plasmid or control plasmid was transfected into HGC27 or SGC7901 cell lines, the apoptosis level of cells was analyzed by flow cytometry, and the expression of cleaved-caspase3 protein was detected by Western Blot. The results showed that overexpression of LEF1-AS1 could reduce cell apoptosis (Figure 6F–I). Therefore, we preliminarily concluded that LEF1-AS1 promoted autophagy in GC cells and inhibited apoptosis in GC cells.

### 2.7. miR-5100 Can Directly Bound to the 3′UTR of DEK and Regulate DEK Expression

**Figure 7:**
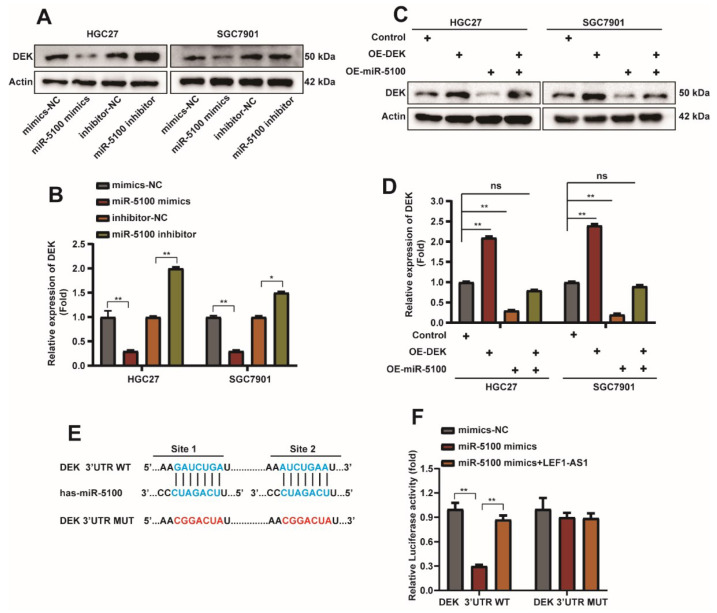
miR-5100 can directly bound to the 3′UTR of DEK and regulate DEK expression. (**A**,**B**) Western Blot detection of DEK expression when miR-5100 mimics/mimics-NC/miR-5100 inhibitor/inhibitor-NC was transfected into HGC27 and SGC7901 cells for 36 h, and the experimental results were quantified with Actin Normalized (*n* = 3, *: *p* < 0.05, **: *p* < 0.01). (**C**,**D**) Western blot detection of DEK protein when miR-5100 mimics + OE-NC/OE-DEK + mimics-NC/5100 mimics + OE-DEK were transfected into HGC27 and SGC7901 cells, co-transfected with mimics-NC and OE-NC as controls. Quantitative analysis of experimental results was normalized with Actin (*n* = 3, **: *p* < 0.01). (**E**) Construction of WTDEK- Schematic representation of the 3′UTR-WT and DEK-3′UTR-Mut luciferase reporter plasmids. (**F**) DEK 3′UTR-WT/Mut and mimics-NC/miR-5100 mimics/miR-5100 mimics + LEF1-AS1 were co-transfected, and the luciferase reporter assay verified the targeting between miR-5100 and DEK effect (*n* = 3, **: *p* < 0.01).

To investigate whether the regulation of autophagy and apoptosis of GC cells by miR-5100 is related to DEK, miR-5100 mimics or miR-5100 inhibitor were transfected into HGC27 and SGC7901 cells, respectively. We were find that DEK expression was decreased when miR-5100 was overexpressed, but the miR-5100 inhibitor could upregulate DEK expression (Figure 7A,B and Figure S1D). The rescue experiment results also indicated that miR-5100 was involved in the regulation of DEK expression (Figure 7C,D). It has been reported in the literature that miRNAs induce transcript degradation or inhibit protein translation by specifically binding to the 3′UTR sequences of target mRNAs [47]. It was predicted from the website that miR-5100 could bind to the 3′UTR of DEK. Next, we constructed a luciferase reporter plasmid containing DEK 3′UTR, and constructed a mutant plasmid that mutated the binding sequence of miR-5100 (Figure 7E). DEK 3′UTR-WT/Mut and mimics-NC/miR-5100 mimics/miR-5100 mimics + LEF1-AS1 were co-transfected. The results of the dual luciferase reporter assay showed that miR-5100 could reduce the luciferase activity of DEK, and this inhibition was relieved when the 3′UTR binding site of DEK was mutated. Interestingly, this repression also occurred when miR-5100 was co-transfected with LEF1-AS1, which we will explore below (Figure 7F). The above experimental results suggest that miR-5100 can directly bound to the 3′UTR of DEK and regulate DEK expression.

### 2.8. miR-5100 Reverses the Metastasis Phenotype of GC Induced by Abnormal Expression of DEK

**Figure 8:**
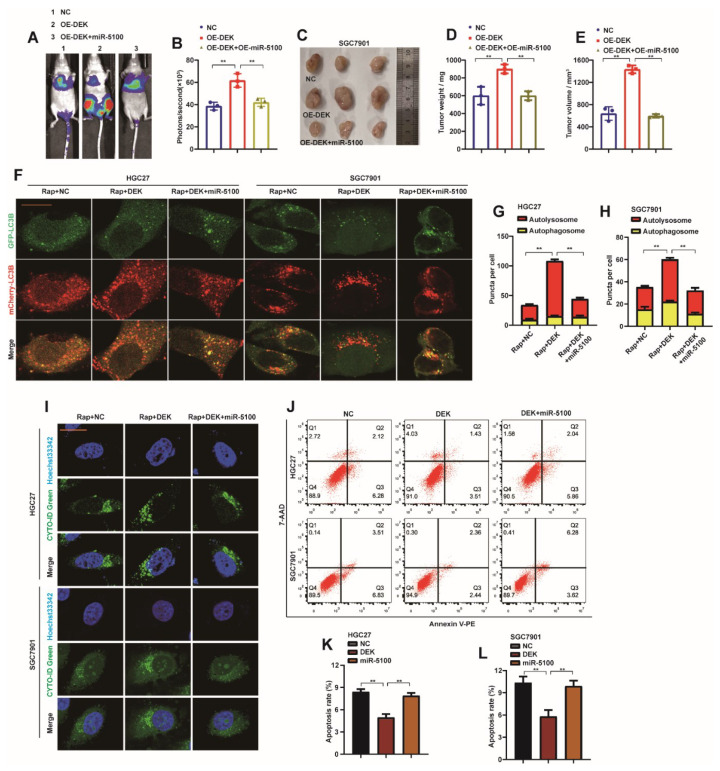
miR-5100 reverses the malignant phenotype of GC induced by abnormal expression of DEK. (**A**,**B**) pre-miR-5100 was introduced into SGC7901-DEK cell lines by lentiviral method, the resulting cell lines were labeled with luciferase, and then injected into nude mice by tail vein to detect the lungs of nude mice Department transfer situation. Statistical analysis of the fluorescence signal intensity was performed (*n* = 3, **: *p* < 0.01). (**C**–**E**) Pre-miR-5100 was introduced in SGC7901-DEK cell line, SGC7901-DEK/SGC7901-DEK + miR-5100 or control cell line was injected subcutaneously into nude mice, and subcutaneous xenografts were harvested and treated. Allograft mass and volume were statistically analyzed (**: *p* < 0.01). (**F**) HGC27-DEK/SGC7901-DEK cells stably expressing mCherry-EGFP-LC3B were introduced with pre-miR-5100, respectively, and cells were treated with 5 nM Rap for 8 h, and the level of autophagic flux was observed under confocal microscopy. (**G**,**H**) Autophagosome (red dots) and Autolysosome (yellow dots) were analyzed (**: *p* < 0.01). (**I**) Pre-miR-5100 was introduced into HGC27-DEK/SGC7901-DEK cells, and the level of autophagy were observed under confocal microscopy after 5 nM Rap treatment for 8 h. (**J**–**L**) Pre-miR-5100 was introduced into HGC27-DEK/SGC7901-DEK cells, and the apoptosis level of the cells was detected by flow cytometry, and the results were statistically analyzed (*n* = 3, **: *p* < 0.01).

To verify that miR-5100 can reverse the metastasis phenotype of GC cells caused by the abnormal expression of DEK, we introduced pre-miR-5100 into SGC7901-DEK cell lines by the lentiviral method. First, we found that up-regulation of miR-5100 expression in SGC7901-DEK cells reversed the strong degree of metastasis of SGC7901-DEK cells in nude mice (Figure 8A,B). The introduction of pre-miR-5100 also abrogated the phenomenon that SGC7901-DEK promoted the growth of subcutaneous xenograft tumor mass and volume in nude mice (Figure 8C–E). In vitro experiments found that the introduction of miR-5100 into HGC27-DEK and SGC7901-DEK cells could abolish the promotion of autophagic flux and autophagy by overexpression of DEK (Figure 8F–H). At the same time, the inhibition of apoptosis by DEK was also relieved (Figure 8I,J). These data further confirmed the regulatory effect of miR-5100 on DEK.

### 2.9. LEF1-AS1 Sponge Adsorbs miR-5100 and Regulates the Expression of miR-5100

**Figure 9:**
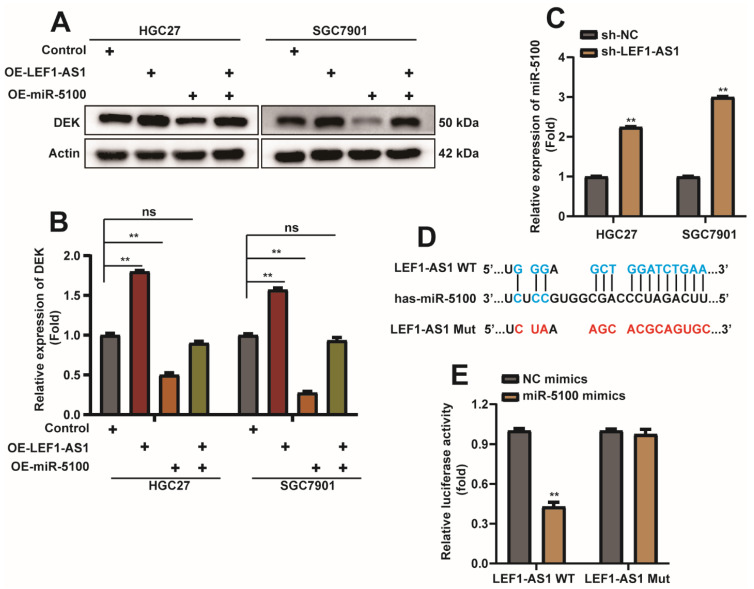
LEF1-AS1 sponge adsorbs miR-5100 and regulates the expression of miR-5100. (**A**,**B**) Western Blot detection of DEK protein when OE-LEF1-AS1 + mimics-NC/miR-5100 mimics + OE-NC/5100 mimics + OE-LEF1-AS1 was transfected into HGC27 and SGC7901 cells, and co-transfected with mimics -NC and OE-NC were used as controls. Quantitative analysis of experimental results was normalized with Actin (*n* = 3, **: *p* < 0.01). (**C**) qPCR was used to detect the expression level of mIR-5100 in HGC27- sh-LEF1-AS1 and SGC7901-sh-LEF1-AS1 cell lines, U6 was used as normalization (*n* = 3, **: *p* < 0.01). (**D**) Schematic diagram of construction of LEF1-AS1-WT and LEF1-AS1-Mut luciferase reporter plasmids. (**E**) LEF1-AS1-WT/Mut and mimics-NC/miR-5100 mimics were co-transfected, and the luciferase reporter assay verified the sponge effect between miR-5100 and LEFAS1 (*n* = 3, **: *p* < 0.01).

We mentioned above that when LEF1-AS1 was co-transfected with miR-5100, the inhibitory effect of miR-5100 on DEK 3′UTR luciferase activity was also relieved (Figure 7F), which aroused our interest. Interestingly, we found that the overexpression of LEF1-AS1 reversed the inhibition of DEK expression by miR-5100 (Figure 9A,B). We examined the expression level of miR-5100 in HGC27-sh-LEF1-AS1 and SGC7901-sh-LEF1-AS1 cells and found that the expression level of miR-5100 increased when the expression of LEF1-AS1 was inhibited (Figure 9C). Recently, lncRNA was found to epigenetically silence miRNA expression at the transcriptional level [48], thereby promoting tumor progression, and the website predicted the binding possibility of miR-5100 to LEF1-AS1. To further explore the relationship between miR-5100 and LEF1-AS1, we constructed a luciferase reporter plasmid containing the LEF1-AS1 sequence, and constructed a mutant plasmid that mutated the sequence bound by miR-5100 (Figure 9D). LEF1-AS1-WT/Mut and mimics-NC/miR-5100 mimics were co-transfected. The results of the dual-luciferase reporter assay showed that miR-5100 could inhibit the luciferase activity of LEF1-AS1, and this inhibition was relieved when the site on LEF1-AS1 that bound to miR-5100 was mutated (Figure 9E). These data illustrate the sponge effect between LEF1-AS1 and miR-5100.

### 2.10. LEF1-AS1 Reverses the Metastasis Phenotype of GC Induced by Abnormal Expression of miR-5100

**Figure 10:**
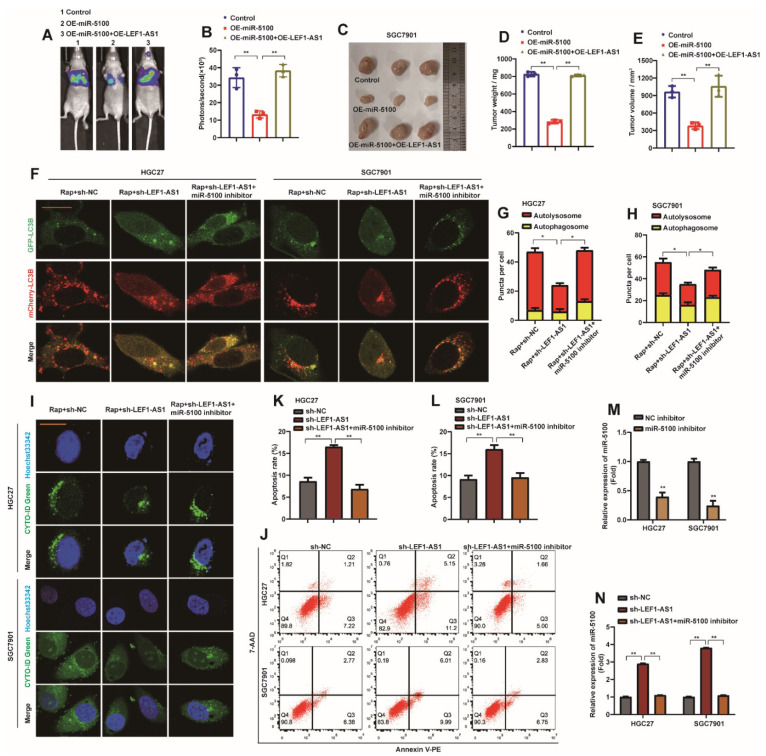
LEF1-AS1 reverses the malignant phenotype of GC induced by abnormal expression of miR-5100. (**A**,**B**) LEF1-AS1 was introduced into SGC7901-miR-5100 cell line by lentiviral method, then the cell line was labeled with luciferase, and then injected into nude mice by tail vein to detect the lung metastasis of nude mice. Statistical analysis of the fluorescence signal intensity was performed (*n* = 3, **: *p* < 0.01). (**C**–**E**) LEF1-AS1 was introduced in SGC7901-miR-5100 cell line, SGC7901-miR-5100/SGC7901-miR-5100 + LEF1-AS1 or control cell line was injected subcutaneously into nude mice, and subcutaneous heterozygous cells were harvested. Tumors were implanted and the mass and volume of the xenografted tumors were statistically analyzed (**: *p* < 0.01). (**F**) HGC27-sh-LEF1-AS1/SGC7901-sh-LEF1-AS1 cells stably expressing mCherry-EGFP-LC3B were transfected with miR-5100 inhibitor, respectively, and cells were treated with 5 nM Rap for 8 h under confocal microscopy The levels of autophagic flux were observed. (**G**,**H**) Autophagosome (red dots) and Autolysosome (yellow dots) were analyzed (*: *p* < 0.05). (I) HGC27-sh-LEF1-AS1/SGC7901-sh-LEF1-AS1 cells were transfected with miR-5100 inhibitor and treated with 5 nM Rap for 8 h to observe the level of autophagy under a confocal microscope. (**J**–**L**) HGC27-sh-LEF1-AS1/SGC7901-sh-LEF1-AS1 cells were transfected with miR-5100 inhibitor, the apoptosis level of cells was detected by flow cytometry, and the results were statistically analyzed (*n* = 3, **: *p* < 0.01).

Similarly, in order to verify that LEF1-AS1 can reverse the metastasis phenotype of GC cells caused by abnormal expression of miR-5100, LEF1-AS1 was introduced into SGC7901-miR-5100 cell lines by lentiviral method. In vivo experiments, we found that up-regulation of LEF1-AS1 could reverse the slow migration of SGC7901-miR-5100 cells in nude mice (Figure 10A,B). It also reversed the phenomenon that SGC7901-miR-5100 reduced the mass and volume of subcutaneous xenografts in nude mice (Figure 10C–E). HGC27-sh-LEF1-AS1 and SGC7901-sh-LEF1-AS1 cells were transfected with miR-5100 inhibitor for in vitro experiments. The results showed that the introduction of miR-5100 inhibitor reversed the inhibitory effect of sh-LEF1-AS1 on autophagic flux and autophagy (Figure 10F–H), and at the same time slowed down the promoting effect of sh-LEF1-AS1 on cell apoptosis (Figure 10I,J). miR-5100 inhibitor was transfected in HGC27 or SGC7901 cells, and the expression of miR-5100 was determined to be inhibited (Figure 10M). When miR-5100 inhibitor was introduced into HGC27-sh-LEF1-AS1 or SGC7901-sh-LEF1-AS1 cells, qPCR detection showed that the promoting effect of sh-LEF1-AS1 on miR-5100 was abolished (Figure 10N). The above data further confirm that the sponge effect between LEF1-AS1 and miR-5100 affects the autophagy and apoptosis of GC cells.

## 3. Discussion

In the present study, we found that DEK is highly expressed in GC tissues and cell lines. Silencing DEK inhibits autophagy and promotes apoptosis. In addition, silencing DEK inhibited the metastasis of GC cells and the growth of subcutaneously transplanted tumors in nude mice. We found that DEK regulates autophagy and apoptosis through the AMPK/mTOR signaling pathway. As an upstream regulator of DEK, miR-5100 inhibits autophagy in GC cells, promotes apoptosis, and regulates DEK expression by targeting the 3′UTR of DEK. In addition, LEF1-AS1 can promote autophagy and inhibit apoptosis of GC cells by sponge-absorbing miR-5100. In conclusion, we discovered a novel regulatory mechanism of LEF1-AS1/miR-5100/DEK, which has important implications for the occurrence and development of GC.

Autophagy is a tightly regulated process of removing cytoplasmic components or damaged organelles that begins with the formation of double-membrane vesicles called autophagosomes [49]. Autophagy plays an important role in the chemoresistance mechanism of GC cells. Aberrantly activated autophagy induced by chemotherapeutics can provide energy to support cancer cells, thereby promoting chemoresistance [50]. Autophagy and apoptosis are two distinct cellular processes with often opposite outcomes, and their signaling pathways are extensively interconnected through various crosstalk mechanisms. Many related proteins regulate autophagy and apoptosis [51].

The oncogene DEK is located on human chromosome 6p22.3 and was originally identified as a fusion to the 3′ portion of the chromosome 9 NUP214 (CAN) gene in a specific subtype of acute myeloid leukemia (AML) patients [52,53]. It has gained high attention in recent years because it is highly expressed and plays a crucial role in tumorigenic events for a variety of such as retinoblastoma [54], glioblastoma [55], bladder cancer [56], colorectal cancer [57], hepatocellular carcinoma (HCC) [58,59], head and neck squamous cell carcinoma (HNSCC) [60], ovarian tumors [61], and other tumor types [5,62,63,64]. It has been shown that patients with early GC with high DEK expression have shorter disease-free survival and overall survival than patients with low expression [6], and it is a potential biomarker related to malignant phenotype in GC tissue and plasma [65]. This paper concluded that the high expression of DEK was associated with the malignant phenotype of GC in the nude mouse lung metastasis model and subcutaneous xenograft model, which was consistent with the literature reports. Hui et al. reported that microRNA-1292-5p inhibited GC cell growth, migration, and invasion by targeting DEK [19]. Wang et al. showed that CD36 upregulates DEK transcription and promotes cell migration and invasion through GSK-3beta/β-catenin-mediated epithelial-mesenchymal transition [18]. In addition, Zhang et al. suggested that MiR-138-5p inhibited GC cell proliferation by targeting DEK [17]. In promoting tumorigenesis and tumor progression, DEK has been shown to promote cell growth and self-renewal, while inhibiting cell differentiation, premature and apoptosis of malignant cells [5,9,66]. In the present study, we found that knockdown of DEK in GC cells inhibited autophagy and promoted cell apoptosis. A recent study reported that DEK-NUP214 fusion protein increased myeloid cell proliferation in AML cells through the mTOR pathway [67]. This suggests that DEK is involved in the mTOR signaling pathway. Excitingly, our experimental results confirm that DEK participates in the autophagy and apoptosis of GC cells through the AMPK/mTOR signaling pathway.

Based on the above promising experimental results, we next explored the molecular mechanism of DEK overexpression in GC. In recent years, the study of miRNAs has played an important role in controlling the development and progression of GC. Such as cell proliferation [68], invasion [69], metastasis [70], tumor growth [71] and apoptosis and drug resistance [72]. Our previous research results show that miR-5100 is lowly expressed in GC cells and can target CAAP1 to regulate autophagy and apoptosis of GC cells [73]. What interested us was that miR-5100 also had the potential to target DEK. We demonstrated that miR-5100 could inhibit the luciferase activity of DEK using a luciferase reporter assay, while overexpression of miR-5100 could down-regulate the expression of DEK. Current research on miR-5100 mainly focuses on lung cancer [23,74,75], pancreatic cancer [29], oral squamous cell carcinoma [76] and prostate cancer [77]. miR-5100 is upregulated during osteoblast differentiation and fine-tunes osteoblast differentiation through the miR-5100/Tob2/osterix network [78]. Increased expression of miR-5100 in non-small cell lung cancer and is associated with poor prognosis [75], promoting lung cancer tumor growth by promoting G1/S transition and targeting Rab6 [79], exosomes-mediated miR-193a-3p, miR-210-3p, and miR-5100 can promote lung cancer cell invasion by activating STAT3 signaling-induced EMT [28]. In the study of miR-5100 regulating the phenotype of pancreatic cancer cells, Chijiiwa et al. found that miR-5100 has inhibitory effects on the occurrence and metastasis of pancreatic cancer [29]. The above conclusions seem to indicate that mIR-5100 may play diametrically opposite roles in different tumors, and the specific role of miR-5100 remains to be further explored.

It has been reported that LEF1-AS1 is oncogene that promotes tumor progression upregulated [80] and is causes retinoblastoma [81]. A study on ovarian cancer showed that the expression of LEF1-AS1 was up-regulated in ovarian cancer tissues, and down-regulation of the LEF1-AS1 gene could inhibit the proliferation, migration, and invasion of ovarian cancer cells. Mechanistically, LEF1-AS1 exerts its oncogenic function by acting as a sponge with miR-1285-3p to inhibit miRNA activity [38]. LEF1-AS1 promotes the malignant behavior of glioblastoma cells, and LEF1-AS1 acts as a ceRNA of miR-543 and positively regulates the expression of EN2. Downregulation of miR-543 led to increased malignant behavior of glioblastoma cells, and downregulation of LEF1-AS1 reversed this phenomenon [82]. Furthermore, Liu et al. found that silencing LEF1-as1 inhibited prostate cancer initiation and progression by blocking LEF1 as a molecular sponge for miR-330-5p [83]. Based on previous studies, Li et al. further demonstrated that LEF1-AS1 promotes prostate cancer angiogenesis [41]. Although LEF1-AS1 plays an oncogenic role in most tumors, it has been found to be a cancer suppressor in myeloid malignancies. It is significantly overexpressed in normal hematopoietic stem cells but is rarely detected in myeloid malignant cells. Cell experiments showed that LEF1-AS1 could inhibit the proliferation of myeloid malignant tumors and play a protective role in the occurrence and development of tumors [84]. Similar to literature reports, our study found that LEF1-AS1 acts as a sponge with miR-5100 in GC, and regulates autophagy and apoptosis in GC cells by inhibiting the action of miR-5100.

## 4. Materials and Methods

### 4.1. Tissue Samples

A total of 15 pairs of cancer tissues and adjacent tissues from patients undergoing gastric cancer surgery were collected from April 2021 to March 2021, and all patients gave informed consent. It was approved by the Ethics Committee of Tongji Hospital, Huazhong University of Science and Technology. The lesion tissue was divided into the cancer tissue group, and the adjacent tissue (≥3 cm away from the lesion) was used as the control during the operation. The patients were aged between 40–65 years. Participants did not receive chemotherapy or radiation therapy prior to surgery. Tissue specimens were frozen in liquid nitrogen immediately after surgical resection. All procedures performed in this study involving human participants were in accordance with the Declaration of Helsinki (as revised in 2013).

### 4.2. Cell Lines

All cells were cultured at 37 °C with 5% CO_2_. Human gastric mucosal epithelial cells (GES1), human GC cells HGC-27, MGC80-3, and human embryonic kidney cells 293T used in the manuscript were all derived from BNCC (BNCC, Beijing, China). Human GC cell SGC7901 and AGS was bought from Wuhan Procell (Procell, Wuhan, China). Among them, GES1 and 293T cells were cultured in 90% DMEM (Gibco, New York, NY, USA) + 10% FBS (Gibco, New York, NY, USA), AGS cultured in 90% DMEM/F12 (meilunbio, Wuhan, China) + 10% FBS (Gibco, New York, NY, USA), and HGC-27, MGC80-3 and SGC7901 cells were cultured in 90% RPMI-1640 (Gibco, New York, NY, USA) + 10% FBS (Gibco, New York, NY, USA).

### 4.3. Quantitative Real-Time PCR (qRT-PCR)

Total RNAs were extracted with RNA extraction kit (CWBIO, Beijing, China) and cDNA was synthesized by reverse transcription kit (Vazyme, Nanjing, China). SYBR Green Master Mix (YEASEN, Shanghai, China) was used in qPCR experiments. The qRT-PCR program was set as follows: 95 °C for 5 min, followed by 40 cycles of 95 °C for 10 s, 60 °C for 20 s, and 72 °C for 20 s. The data obtained were normalized with GAPDH or U6 and relative expressions were calculated using the 2^−ΔΔCT^ method. The primer information required for the qPCR process is presented in Table S1 of Supplementary Materials.

### 4.4. Plasmids

The LEF1-AS1 or DEK overexpression plasmid (OE-DEK/OE-LEF1-AS1) was constructed by inserting the LEF1-AS1 sequence and the DEK coding sequence amplified from the human genome or human cDNA library into the pLVX-EF1α-IRES-Puro vector (Addgene, New York, NY, USA), respectively. The DEK 3′ noncoding sequence/LEF1-AS1 sequence was inserted into pmirGLO (Addgene, New York, NY, USA) to obtain DEK 3′UTR/LEF1-AS1 wild-type luciferase reporter plasmid (DEK 3′UTR-WT/LEF1-AS1-WT). The mutant plasmids were amplified with DEK 3′UTR-WT/LEF1-AS1-WT as templates. miR-5100 overexpression plasmid (OE-miR-5100), DEK, LEF1-AS1 knockdown plasmids (sh-DEK, sh-LEF1-AS1) were purchased from Wuhan TSINGKE Biological Company (TSINGKE, Beijing, China). The packaging plasmids pCMV-VSV-G and pCAG-dR8.9 required for packaging lentivirus were purchased from Beyotime (Beyotime, Shanghai, China).

### 4.5. Transfection

miR-5100 mimic, mimic control, miR-5100 inhibitor, inhibitor control, si-DEK, and si-RNA control were purchased from RiboBio (RiboBio, Guangzhou, China), and cells were transfected according to the instructions of riboFECT CP Transfection Kit (RiboBio, Guangzhou, China). Lipofectamine 2000 (Invitrogen, CA, USA) was used for the transfection of lentiviral packaging plasmids, and was transfected into 293T cells according to the ratio of target plasmid: pCMV-VSV-G: pCAG-dR8.9 = 4:3:1.

### 4.6. Flow Cytometry for Apoptosis

An AnnexinV-PE/7-AAD apoptosis detection kit (BD, NJ, USA) was used for flow cytometry analysis. The treated cells were collected and washed with pre-cooled PBS, and resuspended in binding buffer to reach a cell concentration of 1 × 10^6^/mL. Take 200 μL cell suspension, add 5 μL Annexin V PE and 10 μL 7-AAD, mix and incubate in the dark for 20 min, and add 400 μL binding buffer before flow cytometry analysis. The experimental results were analyzed using FlowJo V10 (Flow Jo LLC, NJ, USA).

### 4.7. Autophagy Assay

The CYTO-ID^®^ Autophagy detection kit (Enzo, New York, NY, USA) was used for the detection of autophagy levels. According to the instructions, the treated cells were stained, fixed, mounted, and then observed under a confocal fluorescence microscope. The stable cell lines obtained by Lenti-mCherry-EGFP-LC3B (Beyotime, Shanghai, China) infection were used for the subsequent detection of autophagic flux. For Spot counting, 40 cells were analyzed by manual blind evaluation. For each cell, randomly select 20–80 clearly distinguishable discrete points from the red channel as regions of interest, and evaluate individual regions of interest for the presence of green fluorescence to determine whether the region of interest is “red + green” or only for “red”.

### 4.8. Western Blot

Total protein samples were run on 12% or 7.5% SDS-PAGE and then transferred to PVDF membranes. After blocking the membrane with 5% skim milk, anti-GAPDH (ABclonal, AC002, 1:5000, Wuhan, China), Actin (ABclonal, AC026, 1:5000, Wuhan, China), cleaved-caspase-3 (CST, 9664S, 1:1000, Boston, MA, USA)), LC3B (CST, 43566S, 1:1000, Boston, MA, USA), p62 (ABclonal, A19700, 1:1000, Wuhan, China), AMPK (CST, 2532S, 1:1000, Boston, MA, USA), p-AMPK (CST, 2535S, 1:1000, Boston, MA, USA), mTOR (CST, 2983S), 1:1000, Boston, USA), p-mTOR (CST, 50081S, 1:1000, Boston, MA, USA), DEK (CST, 29812S, 1:1000, Boston, MA, USA),CD9 (ABclonal, A19027, 1:1000, Wuhan, China), CD81 (ABclonal, A4863, 1:1000, Wuhan, China), CD63 (ABclonal, A19023, 1:1000, Wuhan, China), and the corresponding secondary antibody incubation (all, ABclonal, Wuhan, China). Protein signals were visualized in the imaging system using the ECL chemiluminescence kit (Beyotime, Shanghai, China).

### 4.9. Dual-Luciferase Reporter Analyses

To verify the binding of LEF1-AS1 and miR-5100 sponges, LEF1-AS1-WT/Mut and miR-5100 mimics/mimics-NC were co-transfected for 48 h, and the luciferase activity was detected by a dual-luciferase assay system (Promega, WI, USA). DEK 3′UTR-WT/Mut and mimics-NC/miR-5100 mimics/miR-5100 mimics + LEF1-AS1 were co-transfected to verify that miR-5100 regulates DEK.

### 4.10. BALB/c Nude Mice Animal Models

Animal xenograft tumor model analysis was described previously [73]. Briefly, four-week-old female BALB/c were obtained from Liaoning Changsheng Organisms (Liaoning, China). All nude mice were fed under the SPF environment of the Laboratory Animal Center of Wuhan University of Science and Technology, and were approved by the Laboratory Animal Ethics Committee of Wuhan University of Science and Technology. 5 × 10^6^ SGC7901 cell lines were injected subcutaneously (*n* = 3 per group), and the subcutaneous tumors were harvested 21 days after inoculation. the subsequent statistical analysis was performed. The injected SGC7901 cell line was infected with sh-DEK expressing lentivirus or miR-5100 expressing lentivirus or DEK expressing lentivirus or co-infected with miR-5100 and DEK expressing lentivirus or co-infected with miR-5100 and LEF1-AS1 expressing lentivirus.

To evaluate the effects of DEK, miR-5100, and LEF1-AS1 on GC lung metastasis in vivo, 4-week-old BALB/c was injected into the tail vein of 200 μL PBS containing 2 × 10^6^ SGC7901 cells (*n* = 3 per group). The injected cells were identical to those used in the Animal xenograft tumor model except that they were luciferase-labeled. To confirm the injection was successful, we measured photon flux throughout the whole body of the mice weekly using an IVIS Lumina Series III (Caliper Life Sciences, Boston, MA, USA). After 28 days, BLI analysis was performed on each mouse to monitor lung metastases.

### 4.11. Immunohistochemistry (IHC) and Hematoxylin-Eosin Staining (HE)

For the histological evaluation of patients and mice, all tissues were soaked in 10% buffered formalin for 24 h and then sent to Wuhan Servicebio Technology Co., Ltd. (Wuhan, China) for paraffin embedding and sectioning to 4 μm. For immunohistochemical analysis, the sections were successively deparaffinized, followed by antigen retrieval with citric acid antigen retrieval buffer, 3% hydrogen peroxide solution to block endogenous peroxidase, BSA blocking tissue, antibody incubation, DAB color development, hematoxylin staining, Dehydrated coverslips. Finally, observe and evaluate under the microscope. The reagents required in the immunohistochemical experiment were purchased from Wuhan Servicebio Technology. For HE staining, standard experimental procedures were followed by Wuhan Servicebio Technology Co., Ltd.

### 4.12. Extraction and Identification of Exosomes

miR-5100 was overexpressed in mesenchymal hepatocytes, and the supernatant of the medium was collected after 72 h of culture, and exosomes were extracted and isolated according to the experimental procedure of the exosome extraction kit (YEASEN, 41201ES25, Shanghai, China). Exosomes were quantified with BCA protein quantification kit (YEASEN, 20201ES76, Shanghai, China), and exosome markers were detected by Western blot. Electron microscopy detection was sent to Wuhan servicebio technology Co., Ltd. For operation according to standard protocols.

### 4.13. Bionformatic Analysis

TCGA (https://www.cancer.gov/aboutnci/organization/ccg/research/structural-genomics/tcga, accessed on 19 October 2021) and UALCAN (http://ualcan.path.uab.edu/cgi-bin/ualcan-res.pl, accessed on 19 October 2021) were used to analyze the expression level of DEK in gastric cancer, Targetscan (https://www.targetscan.org/vert_72/, accessed on 21 October 2021) predicts the targeting effect of miR-5100 on DEK, and LncBase Predicted v2 predicts the sponge effect of LEF1-AS1 and miR-5100 (https://diana.e-ce.uth.gr/lncbasev3, accessed on 21 October 2021).

### 4.14. Statistical Analysis

All experiments were validated in three independent replicates. Statistical analysis was performed using GraphPad Prism 9 and SPSS 19.0 software. All data are presented as mean ± SD (standard deviation) from triplicates. *p* values < 0.05 were statistically significant. Statistical analysis was done using paired Student’s *t*-test; * represents *p* < 0.05, ** represents *p* < 0.01, and *** represents *p* < 0.001.

## 5. Conclusions

In our study, we discovered a novel regulatory mechanism of LEF1-AS1/miR-5100/DEK, which has important implications for the occurrence and development of GC. High expression of DEK can aggravate the malignancy of GC, promote autophagy, and inhibit apoptosis through the AMPK/mTOR signaling pathway. miR-5100 regulates the expression of DEK by targeting the 3′UTR of DEK, and has been shown to inhibit autophagy and promote apoptosis in GC cells in vitro. In addition, LEF1-AS1 can promote autophagy and inhibit apoptosis of GC cells by sponge-absorbing miR-5100. The discovery of LEF1-AS1/miR-5100/DEK signaling axis may become a potential target for GC therapy, providing the latest theoretical support for GC therapy.

## Data Availability

The datasets used or analysed during the current study are available from the corresponding author on reasonable request.

## Supplementary Materials

The following supporting information can be downloaded at: https://www.mdpi.com/article/10.3390/ijms23094787/s1.

## Abbreviations

GC: Gastric Cancer, DEK: DNA-binding proteins and the regulator of chromatin structure DEK, miRNA: microRNA, 3′UTR: 3-untranslation region, lncRNAs: Long non-coding RNAs, FBS: Fetal bovine serum, IHC: Immunohistochemistry, HE: Hematoxylin-Eosin staining, qPCR: Quantitative real-time PCR, TCGA: The Cancer Genome Atlas.

## References

[1] Waldum H., Fossmark R. (2021). Gastritis, Gastric Polyps and Gastric Cancer. Int. J. Mol. Sci..

[2] Bray F., Ferlay J., Soerjomataram I., Siegel R.L., Torre L.A., Jemal A. (2018). Global cancer statistics 2018: GLOBOCAN estimates of incidence and mortality worldwide for 36 cancers in 185 countries. CA A Cancer J. Clin..

[3] Li S., Yang X., Li W., Chen Z. (2021). Comprehensive Analysis of E2F Family Members in Human Gastric Cancer. Front. Oncol..

[4] Recio-Boiles A., Babiker H.M. (2022). Gastric Cancer. StatPearls.

[5] Khodadoust M.S., Verhaegen M., Kappes F., Riveiro-Falkenbach E., Cigudosa J.C., Kim D.S., Chinnaiyan A.M., Markovitz D.M., Soengas M.S. (2009). Melanoma proliferation and chemoresistance controlled by the DEK oncogene. Cancer Res..

[6] Piao J., Shang Y., Liu S., Piao Y., Cui X., Li Y., Lin Z. (2014). High expression of DEK predicts poor prognosis of gastric adenocarcinoma. Diagn. Pathol..

[7] Privette Vinnedge L.M., Ho S.M., Wikenheiser-Brokamp K.A., Wells S.I. (2012). The DEK oncogene is a target of steroid hormone receptor signaling in breast cancer. PLoS ONE.

[8] Koleva R.I., Ficarro S.B., Radomska H.S., Carrasco-Alfonso M.J., Alberta J.A., Webber J.T., Luckey C.J., Marcucci G., Tenen D.G., Marto J.A. (2012). C/EBPα and DEK coordinately regulate myeloid differentiation. Blood.

[9] Wise-Draper T.M., Allen H.V., Jones E.E., Habash K.B., Matsuo H., Wells S.I. (2006). Apoptosis inhibition by the human DEK oncoprotein involves interference with p53 functions. Mol. Cell. Biol..

[10] Wang D.M., Liu L., Fan L., Zou Z.J., Zhang L.N., Yang S., Li J.Y., Xu W. (2012). Expression level of DEK in chronic lymphocytic leukemia is regulated by fludarabine and Nutlin-3 depending on p53 status. Cancer Biol. Ther..

[11] Privette Vinnedge L.M., McClaine R., Wagh P.K., Wikenheiser-Brokamp K.A., Waltz S.E., Wells S.I. (2011). The human DEK oncogene stimulates β-catenin signaling, invasion and mammosphere formation in breast cancer. Oncogene.

[12] Ting N.S., Yu Y., Pohorelic B., Lees-Miller S.P., Beattie T.L. (2005). Human Ku70/80 interacts directly with hTR, the RNA component of human telomerase. Nucleic Acids Res..

[13] Bakkenist C.J., Kastan M.B. (2003). DNA damage activates ATM through intermolecular autophosphorylation and dimer dissociation. Nature.

[14] Deutzmann A., Ganz M., Schönenberger F., Vervoorts J., Kappes F., Ferrando-May E. (2015). The human oncoprotein and chromatin architectural factor DEK counteracts DNA replication stress. Oncogene.

[15] Teng Y., Lang L., Jauregui C.E. (2018). The Complexity of DEK Signaling in Cancer Progression. Curr. Cancer Drug Targets.

[16] Fan D., Wang C., Wang D., Zhang N., Yi T. (2021). Circular RNA circ_0000039 enhances gastric cancer progression through miR-1292-5p/DEK axis. Cancer Biomark. Sect. A Dis. Markers.

[17] Zhang W., Liao K., Liu D. (2020). MiR-138-5p Inhibits the Proliferation of Gastric Cancer Cells by Targeting DEK. Cancer Manag. Res..

[18] Wang J., Wen T., Li Z., Che X., Gong L., Jiao Z., Qu X., Liu Y. (2020). CD36 upregulates DEK transcription and promotes cell migration and invasion via GSK-3β/β-catenin-mediated epithelial-to-mesenchymal transition in gastric cancer. Aging.

[19] Hui W., Ma X., Zan Y., Song L., Zhang S., Dong L. (2018). MicroRNA-1292-5p inhibits cell growth, migration and invasion of gastric carcinoma by targeting DEK. Am. J. Cancer Res..

[20] Barbierato M., Zusso M., Skaper S.D., Giusti P. (2015). MicroRNAs: Emerging role in the endogenous μ opioid system. CNS Neurol. Disord. Drug Targets.

[21] Song J.L., Nigam P., Tektas S.S., Selva E. (2015). microRNA regulation of Wnt signaling pathways in development and disease. Cell. Signal..

[22] Nakamura K., Hiyake N., Hamada T., Yokoyama S., Mori K., Yamashiro K., Beppu M., Sagara Y., Sagara Y., Sugiura T. (2021). Circulating microRNA Panel as a Potential Novel Biomarker for Oral Squamous Cell Carcinoma Diagnosis. Cancers.

[23] Yang L., Lin Z., Wang Y., Gao S., Li Q., Li C., Xu W., Chen J., Liu T., Song Z. (2018). MiR-5100 increases the cisplatin resistance of the lung cancer stem cells by inhibiting the Rab6. Mol. Carcinog..

[24] Li B., Xie Z., Li Z., Chen S., Li B. (2016). MicroRNA-613 targets FMNL2 and suppresses progression of colorectal cancer. Am. J. Transl. Res..

[25] Sun C.C., Li S.J., Zhang F., Zhang Y.D., Zuo Z.Y., Xi Y.Y., Wang L., Li D.J. (2016). The Novel miR-9600 Suppresses Tumor Progression and Promotes Paclitaxel Sensitivity in Non-small-cell Lung Cancer through Altering STAT3 Expression. Mol. Ther. Nucleic Acids.

[26] Abdullah L.N., Chow E.K. (2013). Mechanisms of chemoresistance in cancer stem cells. Clin. Transl. Med..

[27] Shi J., Bao X., Liu Z., Zhang Z., Chen W., Xu Q. (2019). Serum miR-626 and miR-5100 are Promising Prognosis Predictors for Oral Squamous Cell Carcinoma. Theranostics.

[28] Zhang X., Sai B., Wang F., Wang L., Wang Y., Zheng L., Li G., Tang J., Xiang J. (2019). Hypoxic BMSC-derived exosomal miRNAs promote metastasis of lung cancer cells via STAT3-induced EMT. Mol. Cancer.

[29] Chijiiwa Y., Moriyama T., Ohuchida K., Nabae T., Ohtsuka T., Miyasaka Y., Fujita H., Maeyama R., Manabe T., Abe A. (2016). Overexpression of microRNA-5100 decreases the aggressive phenotype of pancreatic cancer cells by targeting PODXL. Int. J. Oncol..

[30] Silva A.M., Moura S.R., Teixeira J.H., Barbosa M.A., Santos S.G., Almeida M.I. (2019). Long noncoding RNAs: A missing link in osteoporosis. Bone Res..

[31] Wang P.S., Wang Z., Yang C. (2021). Dysregulations of long non-coding RNAs—The emerging “lnc” in environmental carcinogenesis. Semin. Cancer Biol..

[32] Huang G.Q., Ke Z.P., Hu H.B., Gu B. (2017). Co-expression network analysis of long noncoding RNAs (IncRNAs) and cancer genes revealsSFTA1P and CASC2abnormalities in lung squamous cell carcinoma. Cancer Biol. Ther..

[33] Hou T., Ye L., Wu S. (2021). Knockdown of LINC00504 Inhibits the Proliferation and Invasion of Breast Cancer via the Downregulation of miR-140-5p. Onco Targets.

[34] Wang X., Li X., Lin F., Sun H., Lin Y., Wang Z., Wang X. (2021). The lnc-CTSLP8 upregulates CTSL1 as a competitive endogenous RNA and promotes ovarian cancer metastasis. J. Exp. Clin. Cancer Res. CR.

[35] Chen K., Hou Y., Liao R., Li Y., Yang H., Gong J. (2021). LncRNA SNHG6 promotes G1/S-phase transition in hepatocellular carcinoma by impairing miR-204-5p-mediated inhibition of E2F1. Oncogene.

[36] Gao J., Dai C., Yu X., Yin X.B., Zhou F. (2021). Long noncoding RNA LEF1-AS1 acts as a microRNA-10a-5p regulator to enhance MSI1 expression and promote chemoresistance in hepatocellular carcinoma cells through activating AKT signaling pathway. J. Cell. Biochem..

[37] Kühnl A., Gökbuget N., Kaiser M., Schlee C., Stroux A., Burmeister T., Mochmann L.H., Hoelzer D., Hofmann W.K., Thiel E. (2011). Overexpression of LEF1 predicts unfavorable outcome in adult patients with B-precursor acute lymphoblastic leukemia. Blood.

[38] Zhang Y., Ruan F. (2020). LncRNA LEF1-AS1 Promotes Ovarian Cancer Development Through Interacting with miR-1285-3p. Cancer Manag. Res..

[39] Wang A., Zhao C., Gao Y., Duan G., Yang Y., Fan B., Wang X., Wang K. (2019). LEF1-AS1 contributes to proliferation and invasion through regulating miR-544a/ FOXP1 axis in lung cancer. Investig. New Drugs.

[40] Gao J., Dai C., Yu X., Yin X.B., Zhou F. (2020). LncRNA LEF1-AS1 silencing diminishes EZH2 expression to delay hepatocellular carcinoma development by impairing CEBPB-interaction with CDCA7. Cell Cycle (Georget. Tex.).

[41] Li W., Yang G., Yang D., Li D., Sun Q. (2020). LncRNA LEF1-AS1 promotes metastasis of prostatic carcinoma via the Wnt/β-catenin pathway. Cancer Cell Int..

[42] Xiang C., Zhang Y., Zhang Y., Liu C., Hou Y., Zhang Y. (2020). lncRNA LEF1-AS1 Promotes Proliferation and Induces Apoptosis of Non-Small-Cell Lung Cancer Cells by Regulating miR-221/PTEN Signaling. Cancer Manag. Res..

[43] Zhang X., Wei M., Fan J., Yan W., Zha X., Song H., Wan R., Yin Y., Wang W. (2021). Ischemia-induced upregulation of autophagy preludes dysfunctional lysosomal storage and associated synaptic impairments in neurons. Autophagy.

[44] Guo S., Liang Y., Murphy S.F., Huang A., Shen H., Kelly D.F., Sobrado P., Sheng Z. (2015). A rapid and high content assay that measures cyto-ID-stained autophagic compartments and estimates autophagy flux with potential clinical applications. Autophagy.

[45] Ali Syeda Z., Langden S.S.S., Munkhzul C., Lee M., Song S.J. (2020). Regulatory Mechanism of MicroRNA Expression in Cancer. Int. J. Mol. Sci..

[46] Liang G., Kan S., Zhu Y., Feng S., Feng W., Gao S. (2018). Engineered exosome-mediated delivery of functionally active miR-26a and its enhanced suppression effect in HepG2 cells. Int. J. Nanomed..

[47] Takahashi R.U., Prieto-Vila M., Kohama I., Ochiya T. (2019). Development of miRNA-based therapeutic approaches for cancer patients. Cancer Sci..

[48] Hao N.B., He Y.F., Li X.Q., Wang K., Wang R.L. (2017). The role of miRNA and lncRNA in gastric cancer. Oncotarget.

[49] Mizushima N., Levine B., Cuervo A.M., Klionsky D.J. (2008). Autophagy fights disease through cellular self-digestion. Nature.

[50] Chen H., Yao X., Di X., Zhang Y., Zhu H., Liu S., Chen T., Yu D., Sun X. (2020). MiR-450a-5p inhibits autophagy and enhances radiosensitivity by targeting dual-specificity phosphatase 10 in esophageal squamous cell carcinoma. Cancer Lett..

[51] Rubinstein A.D., Kimchi A. (2012). Life in the balance—A mechanistic view of the crosstalk between autophagy and apoptosis. J. Cell Sci..

[52] von Lindern M., Fornerod M., van Baal S., Jaegle M., de Wit T., Buijs A., Grosveld G. (1992). The translocation (6;9), associated with a specific subtype of acute myeloid leukemia, results in the fusion of two genes, dek and can, and the expression of a chimeric, leukemia-specific dek-can mRNA. Mol. Cell. Biol..

[53] von Lindern M., Breems D., van Baal S., Adriaansen H., Grosveld G. (1992). Characterization of the translocation breakpoint sequences of two DEK-CAN fusion genes present in t(6;9) acute myeloid leukemia and a SET-CAN fusion gene found in a case of acute undifferentiated leukemia. Genes Chromosomes Cancer.

[54] Grasemann C., Gratias S., Stephan H., Schüler A., Schramm A., Klein-Hitpass L., Rieder H., Schneider S., Kappes F., Eggert A. (2005). Gains and overexpression identify DEK and E2F3 as targets of chromosome 6p gains in retinoblastoma. Oncogene.

[55] Kroes R.A., Jastrow A., McLone M.G., Yamamoto H., Colley P., Kersey D.S., Yong V.W., Mkrdichian E., Cerullo L., Leestma J. (2000). The identification of novel therapeutic targets for the treatment of malignant brain tumors. Cancer Lett..

[56] Evans A.J., Gallie B.L., Jewett M., Pond G.R., Squire J.A. (2004). Defining a 0.5-Mb Region of Genomic Gain on Chromosome 6p22 in Bladder Cancer by Quantitative-Multiplex Polymerase Chain Reaction. Am. J. Pathol..

[57] Lin L., Piao J., Gao W., Piao Y., Jin G., Ma Y., Li J., Lin Z. (2013). DEK over expression as an independent biomarker for poor prognosis in colorectal cancer. BMC Cancer.

[58] Le Y., Huang X., Zhang W., Zhao H., Gang W., Lv F., Lei S., Yong T. (2016). Critical role of DEK and its regulation in tumorigenesis and metastasis of hepatocellular carcinoma. Oncotarget.

[59] Qiao M.X., Li C., Zhang A.Q., Hou L.L., Hu H.G. (2016). Regulation of DEK expression by AP-2α and methylation level of DEK promoter in hepatocellular carcinoma. Oncol. Rep..

[60] Adams A.K., Bolanos L.C., Dexheimer P.J., Karns R.A., Wells S.I. (2015). IRAK1 is a novel DEK transcriptional target and is essential for head and neck cancer cell survival. Oncotarget.

[61] Han S., Xuan Y., Liu S., Zhang M., Lin Z. (2010). Clinicopathological significance of DEK overexpression in serous ovarian tumors. Pathol. Int..

[62] Liu X., Qi D., Qi J., Mao Z., Li X., Zhang J., Li J., Gao W. (2016). Significance of DEK overexpression for the prognostic evaluation of non-small cell lung carcinoma. Oncol. Rep..

[63] Ou Y., Xia R., Kong F., Zhang X., Yu S., Jiang L., Zheng L., Lin L. (2016). Overexpression of DEK is an indicator of poor prognosis in patients with gastric adenocarcinoma. Oncol. Lett..

[64] Wang X., Lin L., Ren X., Lin Z., Li Z., Li C., Jin T. (2014). High expression of oncoprotein DEK predicts poor prognosis of small cell lung cancer. Int. J. Clin. Exp. Pathol..

[65] Lee K.F., Tsai M.M., Tsai C.Y., Huang C.G., Ou Y.H., Hsieh C.C., Hsieh H.L., Wang C.S., Lin K.H. (2019). DEK Is a Potential Biomarker Associated with Malignant Phenotype in Gastric Cancer Tissues and Plasma. Int. J. Mol. Sci..

[66] Sammons M., Wan S.S., Vogel N.L., Mientjes E.J., Grosveld G., Ashburner B.P. (2006). Negative regulation of the RelA/p65 transactivation function by the product of the DEK proto-oncogene. J. Biol. Chem..

[67] Sandén C., Ageberg M., Petersson J., Lennartsson A., Gullberg U. (2013). Forced expression of the DEK-NUP214 fusion protein promotes proliferation dependent on upregulation of mTOR. BMC Cancer.

[68] Duan J., Zhang H., Qu Y., Deng T., Huang D., Liu R., Zhang L., Bai M., Zhou L., Ying G. (2016). Onco-miR-130 promotes cell proliferation and migration by targeting TGFβR2 in gastric cancer. Oncotarget.

[69] Cui H., Wang L., Gong P., Zhao C., Zhang S., Zhang K., Zhou R., Zhao Z., Fan H. (2015). Deregulation between miR-29b/c and DNMT3A is associated with epigenetic silencing of the CDH1 gene, affecting cell migration and invasion in gastric cancer. PLoS ONE.

[70] Chen L., Lü M.H., Zhang D., Hao N.B., Fan Y.H., Wu Y.Y., Wang S.M., Xie R., Fang D.C., Zhang H. (2014). miR-1207-5p and miR-1266 suppress gastric cancer growth and invasion by targeting telomerase reverse transcriptase. Cell Death Dis..

[71] Zhang D., Xiao Y.F., Zhang J.W., Xie R., Hu C.J., Tang B., Wang S.M., Wu Y.Y., Hao N.B., Yang S.M. (2015). miR-1182 attenuates gastric cancer proliferation and metastasis by targeting the open reading frame of hTERT. Cancer Lett..

[72] Zhang H., Duan J., Qu Y., Deng T., Liu R., Zhang L., Bai M., Li J., Ning T., Ge S. (2016). Onco-miR-24 regulates cell growth and apoptosis by targeting BCL2L11 in gastric cancer. Protein Cell.

[73] Zhang H.M., Li H., Wang G.X., Wang J., Xiang Y., Huang Y., Shen C., Dai Z.T., Li J.P., Zhang T.C. (2020). MKL1/miR-5100/CAAP1 loop regulates autophagy and apoptosis in gastric cancer cells. Neoplasia.

[74] Li C.Y., Wang Y.H., Lin Z.Y., Yang L.W., Gao S.L., Liu T., Zou B.A., Pan Z.C., Song Z.Q., Liu G. (2017). MiR-5100 targets TOB2 to drive epithelial-mesenchymal transition associated with activating smad2/3 in lung epithelial cells. Am. J. Transl. Res..

[75] Wang T., Liu X., Tian Q., Liang T., Chang P. (2017). Increasing expression of miR-5100 in non-small-cell lung cancer and correlation with prognosis. Eur. Rev. Med. Pharmacol. Sci..

[76] Wei Z., Lyu B., Hou D., Liu X. (2021). Mir-5100 Mediates Proliferation, Migration and Invasion of Oral Squamous Cell Carcinoma Cells Via Targeting SCAI. J. Investig. Surg..

[77] Mello-Grand M., Bruno A., Sacchetto L., Cristoni S., Gregnanin I., Dematteis A., Zitella A., Gontero P., Peraldo-Neia C., Ricotta R. (2021). Two Novel Ceramide-Like Molecules and miR-5100 Levels as Biomarkers Improve Prediction of Prostate Cancer in Gray-Zone PSA. Front. Oncol..

[78] Wang H., Cui Y., Luan J., Zhou X., Li C., Li H., Shi L., Han J. (2017). MiR-5100 promotes osteogenic differentiation by targeting Tob2. J. Bone Miner. Metab..

[79] Huang H., Jiang Y., Wang Y., Chen T., Yang L., He H., Lin Z., Liu T., Yang T., Kamp D.W. (2015). miR-5100 promotes tumor growth in lung cancer by targeting Rab6. Cancer Lett..

[80] Lu X., Qiao L., Liu Y. (2020). Long noncoding RNA LEF1-AS1 binds with HNRNPL to boost the proliferation, migration, and invasion in osteosarcoma by enhancing the mRNA stability of LEF1. J. Cell. Biochem..

[81] He H., Qin M. (2020). Long non-coding RNA LEF1-AS1 is involved in the progression of retinoblastoma through regulating the Wnt/β-catenin pathway. Clin. Exp. Pharmacol. Physiol..

[82] Zeng S., Zhou C., Yang D.H., Xu L.S., Yang H.J., Xu M.H., Wang H. (2020). LEF1-AS1 is implicated in the malignant development of glioblastoma via sponging miR-543 to upregulate EN2. Brain Res..

[83] Liu D., Song L., Liang Q., Hao L., Zhang Z., Han C. (2019). Long noncoding RNA LEF1-AS1 silencing suppresses the initiation and development of prostate cancer by acting as a molecular sponge of miR0 via LEF1 repression. J. Cell. Physiol..

[84] Congrains-Castillo A., Niemann F.S., Santos Duarte A.S., Olalla-Saad S.T. (2019). LEF1-AS1, long non-coding RNA, inhibits proliferation in myeloid malignancy. J. Cell. Mol. Med..

