# Supplementary material for: Long Non-Coding LEF1-AS1 Sponge miR-5100 Regulates Apoptosis and Autophagy in Gastric Cancer Cells via the miR-5100/DEK/AMPK-mTOR Axis
Source: Int J Mol Sci. 2022 Apr 26;23(9):4787. doi: 10.3390/ijms23094787 (PMC9101949; doi:10.3390/ijms23094787)
Supplement: Supplementary file 1 [file ijms-23-04787-s001.zip › ijms-1673000-supplementary.pdf]

Supplementary material

Figure legends

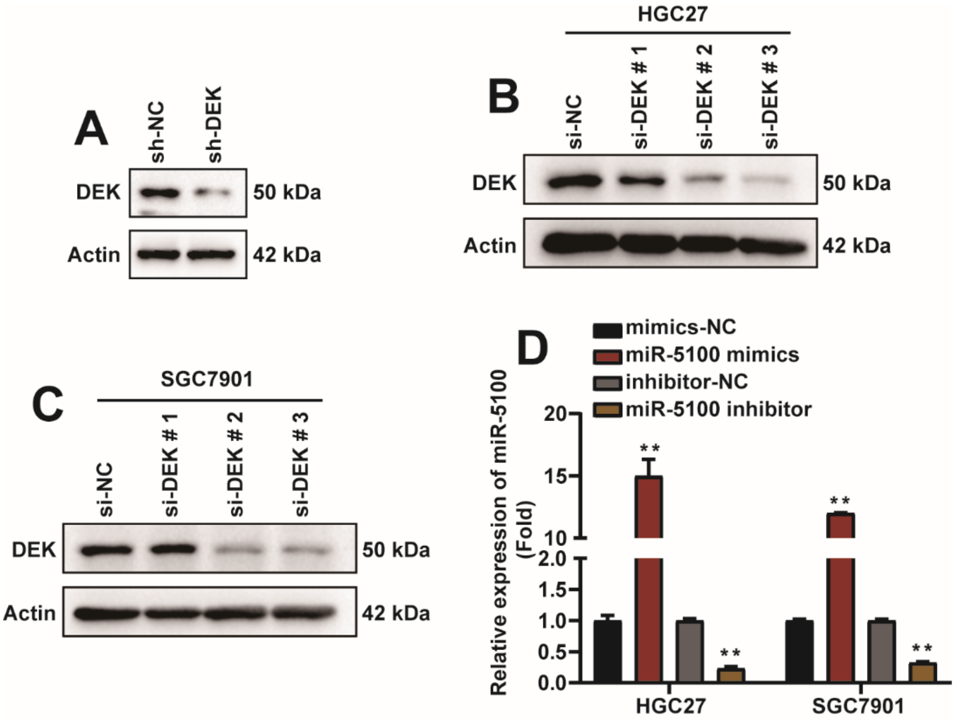

Figure S1. (A) The knockdown efficiency of DEK was detected by Western blot (n=3). **(B and C)** HGC27 or SGC7901 cells were transfected with si-DEK or si-NC, cells were harvested 36 h later, and DEK protein was detected by western blot (n=3). **(D)** miR-5100 mimics or miR-5100 inhibitor were transfected into HGC27 and SGC7901 cells, respectively. After 36 hours, the cells were harvested, and total miRNA was extracted to detect the expression level of miR-5100, which was normalized to U6 (n=3, $^{**}P<0.01$).

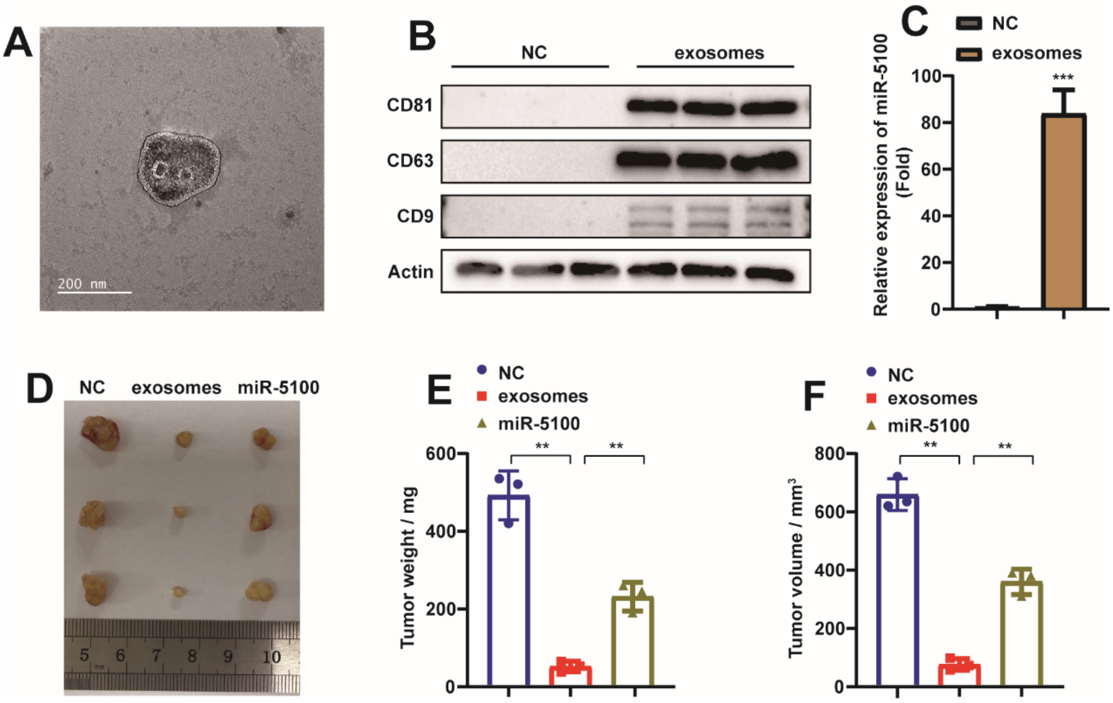

Figure S2. (A) miR-5100 was overexpressed in mesenchymal stem cells, and the cell suspension was harvested for exosome extraction according to standard procedures, and the exosomes were observed under an electron microscope. **(B)** Western blot detection of exosome markers. **(C)** High expression of miR-5100 was detected in exosomes. **(D, E and F)** Nude mice were injected subcutaneously with the same number of SGC7901 cells (1×10^7). Nude mice were randomly divided into 3 groups after successful subcutaneous tumor modeling. PBS (100 µl)/exosomes (0.5 mg/kg in 100 µl PBS)/miR-5100 liposomes (0.5 mg/kg in 100 µl PBS) were injected into nude mice via tail vein for daily treatment, and the subcutaneous tumors were harvested after four weeks and the mass and volume were counted (**P<0.01).

Table S1. Primer information required for qPCR process.

Primer name	Primer sequence (5′–3′)
qPCR-DEK-Forward	AACTGCTTTACAACAGGCCAG
qPCR-DEK-Reverse	ATGGTTTGCCAGAAGGCTTTG
qPCR-miR-5100-Forward	GTACCGTTCTCCGTGGCGACC
qPCR-miR-5100-Reverse	AGTGCAGGGTCCGAGGTATTC
qPCR-Actin-Forward	CATGTACGTTGCTATCCAGGC
qPCR-Actin-Reverse	CTCCTTAATGTCACGCACGAT
qPCR-GAPDH-Forward	CACCATTGGCAATGAGCGGTTC
qPCR-GAPDH-Reverse	AGGTCTTTGCGGATGTCCACGT
qPCR-U6-Forward	CTCGCTTCGGCAGCACA
qPCR-U6-Reverse	AACGCTTCACGAATTTGCGT
